# Epitaxy of III-Nitrides on β-Ga_2_O_3_ and Its Vertical Structure LEDs

**DOI:** 10.3390/mi10050322

**Published:** 2019-05-13

**Authors:** Weijiang Li, Xiang Zhang, Ruilin Meng, Jianchang Yan, Junxi Wang, Jinmin Li, Tongbo Wei

**Affiliations:** 1 State Key Laboratory of Solid-State Lighting, Institute of Semiconductors, University of Chinese Academy of Sciences, Beijing 100083, China; wjli18@semi.ac.cn (W.L.); zhangxiang@semi.ac.cn (X.Z.); mengruilin@semi.ac.cn (R.M.); yanjc@semi.ac.cn (J.Y.); jxwang@semi.ac.cn (J.W.); jmli@semi.ac.cn (J.L.); 2 Center of Materials Science and Optoelectronics Engineering, University of Chinese Academy of Sciences, Beijing 100049, China; 3 Beijing Engineering Research Center for the 3rd Generation Semiconductor Materials and Application, Beijing 100083, China

**Keywords:** β-Ga_2_O_3_, III-Nitrides, monoclinic, hexagonal arrangement, high-power, current distribution, vertical structure LED

## Abstract

β-Ga_2_O_3_, characterized with high n-type conductivity, little lattice mismatch with III-Nitrides, high transparency (>80%) in blue, and UVA (400–320 nm) as well as UVB (320–280 nm) regions, has great potential as the substrate for vertical structure blue and especially ultra violet LEDs (light emitting diodes). Large efforts have been made to improve the quality of III-Nitrides epilayers on β-Ga_2_O_3_. Furthermore, the fabrication of vertical blue LEDs has been preliminarily realized with the best result that output power reaches to 4.82 W (under a current of 10 A) and internal quantum efficiency (IQE) exceeds 78% by different groups, respectively, while there is nearly no demonstration of UV-LEDs on β-Ga_2_O_3_. In this review, with the perspective from materials to devices, we first describe the basic properties, growth method, as well as doping of β-Ga_2_O_3_, then introduce in detail the progress in growth of GaN on (1 0 0) and (−2 0 1) β-Ga_2_O_3_, followed by the epitaxy of AlGaN on gallium oxide. Finally, the advances in fabrication and performance of vertical structure LED (VLED) are presented.

## 1. Introduction

Due to their unique and very desirable properties, such as the direct tunable wide bandgap that span the infrared, ultraviolet, and whole visible spectra region, high chemical and thermal stability, and high mobility, III-Nitrides materials (InN, GaN, and AlN) have attracted worldwide attention both in research and industry. Applications of III-Nitrides in photoelectronic devices and high-power electronic devices, such as laser diodes (LDs), visible and ultraviolet (UV) detectors, and light emitting diodes (LEDs), have been realized [1,2,3,4,5,6]. LEDs therein are the most mature devices with high brightness, compact structure, low energy consumption, high switching speed, long lifetime, and environmental friendliness.

GaN-based blue-LEDs were first commercially introduced in the 1990s [7]. Over the past two decades, due to the huge advances in the crystalline quality of materials and configuration of devices, a wall-plug efficiency reaching around 80% has been realized in industry [8], which has led to a massive market of GaN-based solid-state lighting. Compared with traditional mercury-based UV sources, Al_x_Ga_1-x_N (x from 0 to 1) based UV-LEDs are more attractive for potential applications of non-line-of-sight communications, water purification, food or medical equipment sterilization, phototherapy, UV curing, detection as well as identification of biological or chemical agents, plant growth lighting, and so on [9,10,11,12,13,14,15,16]. Rapid progress in the development of III-Nitrides based UV-LEDs has been forecasted. Yole Développement predicted that there will be an annual growth rate of more than 28% for UV-LED components in the world market, and the aggregate volume will reach $520 million US dollars by 2019 [17].

Up to now, despite the fact that plentiful efforts have been made, there is still a challenge to obtain high-power III-Nitrides based LEDs, especially in the UV region [18,19,20]. The efficiency of LEDs will decrease with increased injection current density, which is the so called efficiency droop effect. Therefore, high-power LEDs are still beyond realization and cannot completely replace the traditional illumination techniques due to the degraded efficiency and rising cost when it comes to high-power applications [1,2,21,22].

The efficiency droop effect can be ascribed to various reasons, including the self-heating effect, carrier delocalization, electron overflow due to the different effective masses of electrons and holes, auger recombination due to the high concentration of carriers, and poor hole injection due to the poor p-type doping efficiency [3,4,5,6,7,8,23,24,25,26,27,28]. It is worth noting that the current density is defined as dividing injection current by chip area, which is not the authentic value when the injection current localizes in a portion of the chip. Consequently, the electron overflow and auger recombination will take place earlier due to the current crowding effect. An earlier overflow and Auger recombination may lead to an earlier self-heating effect that further decrease the output power of LEDs. Thus, uniformizing the current distribution in the active region is of great significance to inhibit the droop effect and improve the performance of devices [11,29].

The widely used sapphire (Al_2_O_3_) substrate has large lattice mismatch (14% with GaN) and large thermal expansion coefficient mismatch (30% with GaN) with III-Nitrides materials [30,31,32], which will introduce high threading dislocation density (TDD) [32,33] acting as nonradiative recombination and scattering centers that deteriorate the performance of LEDs [34]. Furthermore, LEDs are fabricated in a horizontal structure since the sapphire substrates are insulated, leading to that both the n- and p-contact must be established at the top surface of the devices. In this way, the total emission area is reduced, and the fabrication, the encapsulation, as well as the integration procedure become more complex. Moreover, the efficiency droops rapidly with an increased current as the aftermath of current crowding and poor heat distribution due to lateral injection.

Vertical structure is prospective to overcome the problems mentioned above for LEDs fabricated on sapphire [35]. Due to its straightforward configuration, the series resistance of vertical structure LEDs (VLEDs) is less than that of conventional LEDs. Thus, lower forward operating voltage of VLEDs can be obtained, leading to a reduced thermal load. Doan et al. found that the forward I–V curves of GaN-based VLEDs was steeper compared with the lateral structure, indicating a reduced series resistance [36]. A forward voltage of vertical LEDs around 4.5 V at 200 mA compared to 5.8 V for lateral LED on sapphire was reported by Cao et al. [37]. Also, the reduced thermal resistance (Rth) of GaN-based VLEDs can lead to an enhanced heat dissipation ability. Doan et al. demonstrated that the Rth of GaN-based LEDs could be reduced by 55% employing a vertical configuration [38]. A vertical path of current injection can result in a more uniform current distribution, avoiding the localized overheating. Theoretical calculation performed by Li et al. manifested that the injected current of lateral LEDs mostly concentrated in the region below the p electrode, while it was more dispersive for vertical LEDs [39]. In addition, given that there is only one electrode on both the top and bottom sides of VLEDs, the light extraction efficiency can be improved due to the decreased absorption. Additionally, the simplified structure of VLEDs can lower the cost of device fabrication. All these advantages together make it possible for vertical LEDs to work at a high injection current. The GaN-based VLEDs reported by Cao et al. showed higher output power compared with conventional LEDs [37]. A delayed saturation of output power with increased current was obtained due to the reduced efficiency droop effect of VLEDs. The optical degradation of VLEDs was less than 5% after stress compared to 12% for lateral LEDs, indicating an improved reliability with vertical configuration. Thus, vertical structure provides a potential way to realize high brightness and high power LEDs.

So far there are two prevalent methods to manufacture vertical LEDs, one of which is the lift-off and wafer-bonding technique. The usual approach for separating the III-Nitrides epilayer from the sapphire substrate is the laser lift-off (LLO) technique [40], which will, however, introduce a rough surface to the epilayer and substrate due to the physical and thermal impact [41,42]. Recently, the chemical lift-off (CLO) technique for GaN has attracted much attention due to its excellent surface morphology [43]. Materials (e.g., CrN, ZnO, carbon nanotube and Ga_2_O_3_ [43,44,45,46,47,48]), which are successfully employed in the growth of GaN as not only a buffer layer but also a sacrificial layer, have been reported. The CLO process offers a possible approach for III-Nitrides-based vertical structure LEDs. However, it is noteworthy that both the LLO and CLO methods will increase the complexity of fabrication, as will the cost. On this ground, the direct growth of III-Nitride semiconductors on conductive substrates are preferable. The commonly used conductive substrates in vertical LEDs include Si, GaN, and SiC [49]. Silicon is currently the most widely used semiconductor material due to its low cost, large size (6–12 inches), high quality, and a high thermal conductivity compared with sapphire. However, due to the large lattice mismatch (16.9% with GaN) and thermal mismatch (57% with GaN) between Si and III-Nitrides [50], the epilayer will generate a large number of defects, and even cracks, making it quite difficult to grow high-quality III-Nitrides films on silicon substrates. Moreover, silicon is non-transparent in the whole region of UV spectra, deteriorating the light extraction efficiency of UV-LEDs on silicon. SiC has poly type structures including cubic phase (3C-SiC), hexagonal phase (2H-SiC, 4H-SiC, 6H-SiC), and rhombic phase (15R-SiC). Among the substrates mentioned above for heteroepitaxy of III-Nitrides, 6H-SiC exhibits the smallest lattice mismatch (3.4%) and thermal mismatch with GaN, leading to a better crystalline quality of epilayers on SiC than that on silicon [51]. Compared with the GaN layer, a lower refractive index (2.65) of SiC can improve the light extraction efficiency due to the suppressed total internal reflection. Also, SiC has a thermal conductivity of 3–5 W/cm·K, which is three times that of silicon, and can furthermore enhance the heat dissipation and inhibit the self-heating effect of VLEDs. However, an absorption edge of 380 nm will limit the application of SiC as a substrate for vertical LEDs in the deep UV region. The high cost also impedes the extensive use of SiC. Homoepitaxy of GaN can be realized using GaN itself as a substrate. Due to the elimination of lattice mismatch and thermal mismatch, ultrahigh crystalline quality and ultralow density of threading dislocations can be obtained for GaN. In addition, the lattice mismatch between the GaN substrate and AlGaN alloy is also quite low (between 0% and 2.4%, depending on Al-molar fraction). However, being similar to the SiC substrate, an absorption edge of 365 nm, and a high cost, will act as obstacles to the application of GaN substrate, especially in deep UV spectra. Thus, the exploration of novel material as a conductive substrate for VLEDs with a low cost to prepare and high transmittance in visible, especially UV, region is quite necessary.

Recently, due to its unique properties, β-phase gallium oxide semiconductor attracts more and more attention in various fields. β-Ga_2_O_3_ based power electronic devices, including Schottky diodes and metal-semiconductor field-effect transistors have been reported, and photoelectronic devices including x-ray detectors and solar-blind deep-ultraviolet Schottky photodetectors with bulk-like, film-like, as well as nanowire-like gallium oxides, also have been carried out [52,53,54,55]. In addition, β-Ga_2_O_3_ based gas sensors for H_2_, O_2_, CO, or CH_4_ detection, photocatalytic devices for water splitting and gas degradation, and Er doped β-Ga_2_O_3_ for photoresponse or luminescence have been demonstrated [56,57,58,59,60,61,62]. Single crystal bulk β-Ga_2_O_3_ has a wide bandgap of 4.8 eV and an absorption edge of 260 nm, resulting in a high transmittance in visible (>80%) [49], UVA, and UVB region. A high n-type conductivity of β-Ga_2_O_3_ with resistivity of 0.02 Ω·cm can be realized via doping [45]. Also, a low lattice mismatch between β-Ga_2_O_3_ and GaN has been presented [49]. Thus, combined with the advantages of sapphire and SiC, β-Ga_2_O_3_ is a promising candidate as the substrate of vertical structure LEDs.

In this review, we first introduce the properties, the growth method, as well as the doping of β-Ga_2_O_3_, and describe the epitaxial relationship between III-Nitrides and β-Ga_2_O_3_. Then the progresses in epitaxy of GaN on (1 0 0) and (−2 0 1) β-Ga_2_O_3_ substrates are discussed, followed by preliminary attempts to grow AlGaN alloys on gallium oxide. Subsequently, the advances in vertical structure LEDs are introduced. Finally, we present a brief conclusion of the development of vertical blue, especially ultra violet, LEDs on β-Ga_2_O_3_ substrate.

## 2. Properties and Growth Method of Bulk β-Ga_2_O_3_

### 2.1. Structure and Properties

**Figure 1:**
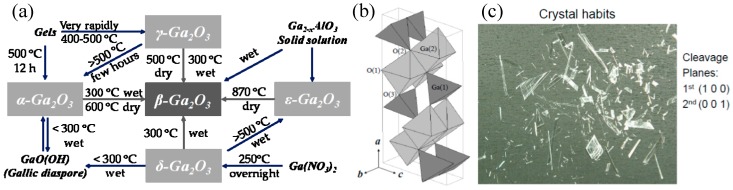
(**a**) Transformation relationships among Ga_2_O_3_ in different crystalline phases and their hydrates. (**b**) Structural schematic illustration of the β-Ga_2_O_3_ unit cell, manifesting the two gallium locations and three oxygen locations. (**c**) Cleavage nature of single crystal β-Ga_2_O_3_. Reprinted with permission from reference [64]. Copyright 2014 SPIE.

In 1952, various polymorphs of gallium oxide including α, β, γ, δ, ε, and a transient κ phase, were experimentally demonstrated through performing research on phase equilibration in the Al_2_O_3_-Ga_2_O_3_-H_2_O system [63]. Among them, β-Ga_2_O_3_ is the most stable one under different conditions. Compared with other polymorphs, β-Ga_2_O_3_ is the only stable one at any temperature below the melting point [64]; other polymorphs of gallium oxide will transform to the beta phase due to their metastability above 750–900 °C, as shown in Figure 1a [65]. The crystal structure of β-Ga_2_O_3_, which belongs to the monoclinic system and the space group C2/m ($C_{2h}^{3}$), was first reported in 1960 [66]. The lattice parameters of β-Ga_2_O_3_ are a = 12.21 Å, b = 3.04 Å, c = 5.80 Å, and β = 103.8°. Figure 1b shows the unit cell of β-Ga_2_O_3_, which contains four formula units occupied by two inequivalent Ga sites and three inequivalent O sites. Two Ga cations are characterized with tetrahedral and octahedral positions, respectively. Ga(I) tetrahedral formula units connect with others only by the corners, while Ga(II) octahedral formula units do it with the edges [64]. As for O anions, three crystallographically inequivalent sites are labeled as O(I), O(II), and O(III), respectively. Two of them are in trigonal coordination and the rest are in tetrahedral coordination. The symmetry of β-Ga_2_O_3_ is quite low with only a 2-fold symmetry along the *b*-axis. Unlike III-Nitrides, the existence of a symcenter leads to the inexistence of spontaneous polarization and piezoelectric polarization for β-Ga_2_O_3_. As illustrated in Figure 1c, β-Ga_2_O_3_ has a nature of cleavage of two planes including the primary (1 0 0) and the subordinate (0 0 1) due to the weak bond. Thus, β-Ga_2_O_3_ usually cracks into needles or plates as a result of fragility.

β-Ga_2_O_3_ is a wide bandgap (4.8 eV) [67], oxide semiconductor characterized with an absorption edge of 260 nm, leading to a remarkable transmittance in the visible (more than 80%), UVA, and UVB range [49]. It has stable chemical natures, such as strong acid as well as alkali resistance, high mechanical strength, and is stable at any temperature below the melting point of 1725 °C. In addition, it has a Vickers hardness of 12.5 GPa along (−2 0 1) plane and a density of 5.95 g/cm^−3^. Due to its low symmetry, the thermal conductivity of β-Ga_2_O_3_ manifests a significant anisotropy, which is 13.6 W/mK along [1 0 0], 22.8 W/mK along [0 1 0], 13.3 ± 1.0 W/mK along [−2 0 1], 14.7 ± 1.5 W/mK along [0 0 1], and 27.0 ± 2.0 W/mK along [1 1 0] [68]. An intentional doping of β-Ga_2_O_3_ will decrease its thermal conductivity resulting from an increased phonon-impurity scattering.

### 2.2. Growth Methods

As mentioned above, β-Ga_2_O_3_ is stable under the whole range of temperature below the melting point, leading to single crystal bulk β-Ga_2_O_3_ that can be prepared by melt growth with a high growth rate, controllable size, and high quality, which also means it can be cheap to grow and process in the future [49,68]. However, there are two main challenges to produce single crystal bulk β-Ga_2_O_3_. One is the quite high melting point. The other is, as the following chemical equations show, the decomposition of β-Ga_2_O_3_ in insufficient oxygen ambient at elevated temperature, which will become more severe above 1200 °C as reported by Ueda et al. [69]:

Ga_2_O_3_ (l,s) → 2GaO (g) + 1/2O_2_ (g),

2GaO (g) → Ga_2_O (g) + 1/2O_2_ (g),

Ga_2_O (g) →2Ga (g) + 1/2O_2_ (g).

**Figure 2:**
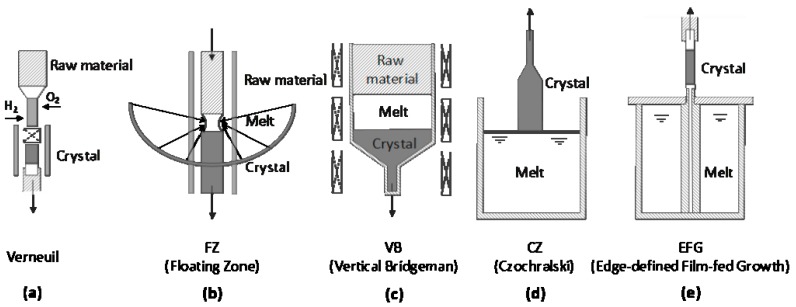
Schematic illustrations of melt growth of β-Ga_2_O_3_: (**a**) Verneuil method; (**b**) floating zone method; (**c**) vertical Bridgeman method; (**d**) Czochralski method; (**e**) edge-defined film-fed growth method.

Figure 2 illustrates the current techniques of melt growth for β-Ga_2_O_3_, including the Verneuil method, floating zone (FZ) method, Czochralski (CZ) method, vertical Bridgeman (VB) method, and edge-defined film-fed growth (EFG) method [70,71,72,73,74,75,76,77,78,79,80]. In 1902, Auguste Verneuil first developed the Verneuil method. As shown Figure 2a, the Verneuil method uses powders as raw materials, and melts them via an oxyhydrogen flame. Then the melting powders cool down and crystallize on the seed crystal. A β-Ga_2_O_3_ single crystal prepared by the Verneuil method was first reported by Chase in 1964 with a size of 3/8 inch in diameter and 1 inch in length [70]. The growth of β-Ga_2_O_3_ using this method was mainly along the *b*-axis. Lorenz et al. demonstrated that single crystal gallium oxide could be prepared under oxidizing conditions, whereas a reducing growth condition would lead to a blue conducting crystal [71]. When doped with Mg and Zr, the single crystal β-Ga_2_O_3_ showed colorless and light blue, respectively [81]. The main disadvantage of the Verneuil method is the inadequate size of as-grown crystals, which is too small for various applications.

The floating zone method employs a high-frequency coil to heat a partial region of the rod-like polycrystalline raw materials, which will suspend on the crystals via surface tension after melting, as shown in Figure 2b. Then the growth of crystals is realized by the upward movement of the coil. Being free of crucible, high purity of β-Ga_2_O_3_ can be obtained due to the reduction of contaminations. The possibility of introducing an oxidizing ambient during the crystal growth by the FZ method can inhibit the decomposition of β-Ga_2_O_3_. In view of the stability of the melt zone, it is difficult to obtain crystals with a large size, and cracks may generate during the cooling down process due to thermal stress. Ueda et al. employed an ordinary ceramic process to prepare the feed rods and realized the Sn doping of β-Ga_2_O_3_ by pressing the SnO_2_ powder into the rods [69]. In 2001, Tomm et al. demonstrated the FZ method growth of β-Ga_2_O_3_ with a growth speed of 5 mm/h. The undoped and Sn-doped crystals were colorless, while the Ti-doped one was pale purple [82]. Crystals with a size of 1 inch along the (1 0 0), (0 1 0), and (0 0 1) axes prepared by the FZ method were demonstrated by Villora et al. in 2004 [72]. In 2006, Zhang et al. reported a FZ method-grown single crystal β-Ga_2_O_3_ along (0 1 0) with a diameter of 6 mm and a length of 20 mm [73].

As shown in Figure 2c, the vertical Bridgeman method uses a crucible to contain the raw material, the melt, as well as the crystal. The raw material is melted through a radio-frequency induction heating furnace. By moving the crucible, while fixing the thermal field in a crystal furnace, the directional solidification process will lead to the growth of β-Ga_2_O_3_ single crystal on seeds. The commonly used material for crucible is an alloy of platinum and rhodium (70% and 30%), which is stable under oxidizing conditions. The main advantages of the VB method are the controllable shape and stoichiometry of crystals due to the use of crucible, which will, however, introduce impurities into the as-grown crystals. Hoshikawa et al. demonstrated the VB growth of β-Ga_2_O_3_ with a diameter of 25 mm along the direction perpendicular to (1 0 0) plane [74]. The seed-free preparation of crystals was realized without adhesion to the crucible wall. Also, the growth of β-Ga_2_O_3_ with different crucible shapes, including a full-diameter type and a conical type, was studied. Ohba et al. characterized the defects of β-Ga_2_O_3_ single crystals, and found no regions of high dislocation density existed near the wafer edge due to the lack of adhesion [75]. In addition, low mean density of dislocation was obtained, which could be ascribed to low thermal gradient during the directional solidification growth of the VB method.

The Czochralski method uses a seed crystal dipping into the melted source materials in a crucible. When pulling and rotating the seed, the reduction of temperature induces the phase transition from liquid state to solid state at the interface between the seed and the melt, as shown in Figure 2d. Due to the high stability of the liquid melt, a large size crystal can be obtained using the CZ method. Tomm et al. first demonstrated the CZ growth of a β-Ga_2_O_3_ single crystal with a growth rate of 2 mm/h [76]. A modified ambient of 10% CO_2_ and 90% Ar gas mixture was found to suppress the evaporation of melted β-Ga_2_O_3_ as CO_2_ would decompose and provide oxygen partial pressures. Galazka et al. grew a 2-inch β-Ga_2_O_3_ single crystal using a CO_2_-containing ambient [77]. High crystalline quality was obtained with the rocking curve FWHM (full width half maximum) values less than 50 arcsec. Dislocations were found to mostly propagate parallel to (1 0 0) plane. In addition, Galazka et al. further studied the segregation of dopants in β-Ga_2_O_3_ single crystal prepared by the CZ method and their influence of optical properties [83]. Dopants such as Ce and Al were found to have a thermodynamically stabilizing effect during the growth of crystals by inhibiting decomposition. The doping of Ce would not degrade the transparency of β-Ga_2_O_3_, while the doping of Cr would lead to three more absorption bands. When doped with Al, the absorption edge of β-Ga_2_O_3_ would shift due to the formation of Ga_2(1-x)_Al_2x_O_3_.

Edge-defined film-fed growth method is the most prevalent technique for β-Ga_2_O_3_ single crystal preparation. As illustrated in Figure 2e, compared with the CZ method, EFG method additionally employs a shaper or a die located in the crucible. Through the channel, the capillarity will force the transport of melt from the crucible to the top surface of the shaper. The melt will spread out until the edge of the shaper. Thus, the shape and the size of EFG crystals can be precisely controlled. The main advantages of the EFG method are the high speed of growth and the possibility of preparing complicated shapes, while the main disadvantages are the geometry and material of the shaper. In 2008, Aida et al. first reported the EFG of β-Ga_2_O_3_ crystal with a rocking curve FWHM value of 70–160 arcsec. Typically, the as-grown crystals had a length of 70 mm, a width of 50 mm, and a thickness of 3 mm with a growth reaching to 10 mm/h [84]. Mu et al. employed a gas mixture of 50% Ar and 50% CO_2_ to grow a high crystalline quality 1-in β-Ga_2_O_3_ with a rocking curve FWHM value of 43.2 arcsec [85]. Kuramata et al. succeeded in obtaining a large size, including 4-in and 6-in, β-Ga_2_O_3_ single crystals by the EFG method [78,79]. Silicon was found to be the main residual impurity for the unintentionally doped EFG crystals, and the observation of etching pits manifested a dislocation density of 10^3^ cm^−3^. By using an ambient of 20% Ar and 80% CO_2_, the crystalline quality of β-Ga_2_O_3_ prepared by the EFG method was further improved by Zhang et al. with FWHM of rocking curve reaching to 19.06 arcsec [80].

### 2.3. Conductivity Control and Doping

A controllable conductivity for semiconductors is the key to various applications. Thus, the realization of both n-type and p-type β-Ga_2_O_3_ is of great importance. In this section, the doping techniques for β-Ga_2_O_3_ are introduced.

#### 2.3.1. N-Type β-Ga_2_O_3_

β-Ga_2_O_3_ is an intrinsic insulator due to its wide bandgap. However, through modifying the melt growth ambient, n-type conductivity can be obtained [86,87]. Single crystal β-Ga_2_O_3_ prepared by Lorenz et al. using the FZ method was insulated under oxidizing conditions, while it had a n-type conductivity under reducing growth conditions [71]. This n-type conductivity can be ascribed to the oxygen vacancies that act as donors after being ionized [86]. Thus, it is believed that there is a strong relation between the conductivity of unintentionally doped β-Ga_2_O_3_ and the existence of oxygen vacancies. Ueda et al. demonstrated a controllable conductivity of gallium oxides ranging from 10^−9^ Ω^−1^·cm^−1^ to 38 Ω^−1^·cm^−1^ by altering the oxygen concentrations in growth ambient [69]. Insulating crystals were obtained when the growth was performed under pure O_2_ with a gas flow rate of 0.2 m^−3^·h^−1^_._ By introducing the nitrogen gas and decreasing the oxygen contents in the ambient, the conductivity of as-grown crystals gradually increased. The conductivity was 0.63 Ω^−1^·cm^−1^ when the O_2_ gas flow rate decreased to 0.05 m^−3^·h^−1^, and reached a maximum value of 38 Ω^−1^·cm^−1^ with a N_2_/O_2_ ratio of 0.4/0.6. A similar tendency of conductivity controlled by growth atmosphere was reported by Galazka et al. [77]. A gas mixture of Ar and CO_2_ or pure CO_2_ was employed to inhibit the evaporation of CZ grown β-Ga_2_O_3_. CO_2_ could provide an oxygen partial pressure due to its decomposition under high temperatures. By increasing the CO_2_ contents from 30% to 50%, the electron concentration was decreased from 10 × 10^17^ cm^−3^ to 0.4–4.8 × 10^17^ cm^−3^.

In addition, intentional doping with other impurities also can influence the conductivity of β-Ga_2_O_3_. The commonly used donors for n-type conductivity include Sn, Si, Ge, Cl, and F [86,88,89]. Due to the different radius, Si and Ge tend to occupy the tetrahedral Ga(I) site, while Sn tends to substitute the octahedral Ga(II) sites. Both Cl and F tend to occupy the O(I) sites. Ueda et al. doped Sn into FZ grown β-Ga_2_O_3_ by pressing SnO_2_ power into the feed rods [69]. A conductivity of 0.96 Ω^−1^·cm^−1^ was measured for Sn-doped β-Ga_2_O_3_ even though the growth ambient was oxygen. Despite large amounts of Sn evaporated during the growth caused by the low efficiency of incorporation, the rest was still enough for n-type doping. FZ grown β-Ga_2_O_3_ crystals doped with Sn reported by Suziki et al. showed an electrical resistivity of 4.27 × 10^−^^2^ cm^−^^2^ and a carrier density of 2.26 × 10^18^ cm^−^^3^ [90]. The doping concentration of SnO_2_ in crystals ranged from 2 to 10 mol%. Due to the evaporation during growth, the actual doping concentration of Sn atoms was in a range of 20–70 ppm.

Silicon is another potential dopant for n-type β-Ga_2_O_3_. Kuramata et al. experimentally studied the correlation between the effective donor concentration (*N*_d_*-N*_a_) and Si concentration of the FZ grown β-Ga_2_O_3_ [78]. The silicon doping was realized by adding SiO_2_ powder into Ga_2_O_3_ powder. It was found that *N*_d_*-N*_a_ for crystals annealing in nitrogen ambient was nearly equal to the Si concentration, while it kept approximately 1 × 10^17^ cm^−3^ when annealed in an oxygen atmosphere and showed no correlation with the Si concentration. Víllora et al. believed that Si was a more efficient donor dopant for β-Ga_2_O_3_ due to the lower vapor pressure of SiO_2_ compared to SnO_2_ and GeO_2_ [91]. By increasing the concentration of Si impurities, the n-type conductivity of β-Ga_2_O_3_ gradually increased from 0.03 Ω^−1^·cm^−1^ to 50 Ω^−1^·cm^−1^, corresponding to a free carrier concentration from 10^16^ cm^−^^3^ to 10^18^ cm^−^^3^. A saturation of carrier concentration was observed at a doping level of 0.2 mol% due to the segregation of the second-phase. The efficiency of incorporation for Si was close to unity at low doping levels, while it was only 5% of the Si atoms in the feed rod. Interestingly, a Si-ion implantation doping technique for β-Ga_2_O_3_ was presented by Sasaki et al. [92]. The implanted silicon ions were activated by annealing crystals under nitrogen ambient at 900–1000 °C. High activation efficiency above 60% was obtained when the injected Si ions were in range of 1 × 10^19^ cm^−^^3^–5 × 10^19^ cm^−^^3^, and it decreased dramatically with an increased Si^+^ concentration. Additionally, an electrical resistance of 1.4 mΩ·cm was measured with a doping concentration of 5 × 10^19^ cm^−^^3^. Recently, Zhou et al. demonstrated the controllable conductivity of FZ grown β-Ga_2_O_3_ employing Nb as dopants [93]. By increasing the Nb doping concentrations, the electrical resistance of β-Ga_2_O_3_ could be changed from 3.6 × 10^2^ Ω·cm to 5.5 ×10^−^^3^ Ω·cm, corresponding to a carrier concentration from 9.55 × 10^16^ cm^−3^ to 1.8 × 10^19^ cm^−3^.

**Figure 3:**
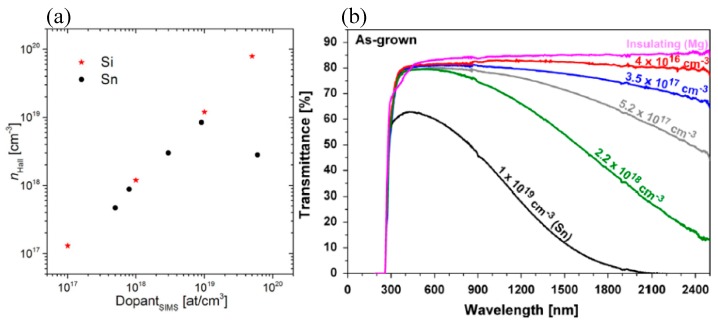
(**a**) Hall free carrier concentration versus the dopant (Si and Sn) concentration obtained by secondary ion mass spectrometry (SIMS). Reprinted with permission from reference [89]. Copyright 2017 The Electrochemical Society. (**b**) Transmittance spectra of β-Ga_2_O_3_ single crystals prepared by the Czochralski (CZ) method with different concentrations of electrons. Reprinted with permission from Reference [77]. Copyright 2014 Elsevier.

Up to now, as illustrated in Figure 3a, the carrier concentrations of n-type β-Ga_2_O_3_ can be highly controlled in a range of 10^16^–10^19^ cm^−3^, and even can reach 10^20^ cm^−3^ by Si doping [89]. Consequently, high conducting β-Ga_2_O_3_ single crystals can be obtained. However, doping will not only change the electrical, but also influence the optical properties of β-Ga_2_O_3_. As shown in Figure 3b, the transmittance of β-Ga_2_O_3_ is decreased with an increasing electron concentration via Sn doping [77]. Insulating β-Ga_2_O_3_ is usually colorless or slightly yellow due to the slight absorption in the blue region, while n-type β-Ga_2_O_3_ shows a bluish-like appearance due to the enhanced absorption of free carriers in red and near infrared regions. Crystals grown in CO_2_ ambient showed grey coloration which could be ascribed to the possible incorporation of carbon impurities. Thus, there should be an eclectic choice between the resistivity and transmittance of β-Ga_2_O_3_, since the crystalline quality and mobility will degrade after doping. Suzuki et al. found that the X-ray rocking curve FWHM value of β-Ga_2_O_3_ was increased from 43 arcsec to 162 arcsec with increased doping concentration of Sn from 32 ppm to 45 ppm [90]. The mobility of free carriers was decreased to 49.3 cm^2^/V·s compared with 87.5 cm^2^/V·s for an undoped sample. Also, an intentional doping of β-Ga_2_O_3_ will decrease its thermal conductivity resulting from an increased phonon-impurity scattering.

#### 2.3.2. P-Type β-Ga_2_O_3_

The lack of an efficient method for p-type conductivity is now one of the major drawbacks of β-Ga_2_O_3_, which largely limits the fabrication of various devices. The difficulty of p-type doping for β-Ga_2_O_3_ can be ascribed to several reasons [94]. Due to the relatively low formation energy of oxygen vacancies, the acceptors doped into crystals can be easily compensated. The high activation energy of acceptors caused by a relatively low energy level of valence band and lack of shallow dopants leads to a poor activation efficiency of dopants. Also, the self-trapping effect and the relatively high effective masses of holes make it harder for the realization of p-type conductivity.

Tomm et al. characterized the electrical properties of FZ grown β-Ga_2_O_3_ doped with Ge and Ti. Hole concentrations of 2 × 10^5^ cm^−3^ and 5 × 10^5^ cm^−3^ were measured for Ge-doped and Ti-doped crystals, respectively, which however, was too low for any practical applications [83]. The study of Mg dopants for β-Ga_2_O_3_ has been carried out by several groups [77,95]. Onuma et al. reported that (0 1 0)-faceted colorless Mg-doped β-Ga_2_O_3_ exhibited a semi-insulating behavior [95]. The electrical resistance measured was around 6 × 10^11^ Ω·cm with Mg concentration in the range of 4 × 10^18^ cm^−3^–2 × 10^19^ cm^−3^. Galazka et al. found that Mg dopants could help to inhibit the formation of spiral for CZ grown β-Ga_2_O_3_ [77]. Insulating crystals were obtained with a doping concentration of Mg above 6 wt ppm. A Mg equilibrium segregation coefficient around 0.10–0.12 at the interface between liquid and solid was estimated. Unlike Sn donors, Mg dopants would lead to a stable insulating behavior of β-Ga_2_O_3_ that was nearly independent with an annealing ambient (oxygen or hydrogen), temperature (up to 1400 °C), time (up to 66 h), and pressure (up to 19 bar).

In addition, other impurities including Zn and N for p-type doping of gallium oxide also have been presented [96,97]. Chang et al. experimentally studied the Zn dopants for Ga_2_O_3_ nanowires using a diffusion doping method [96]. Due to the similar radius (0.074 nm for Zn^2+^ and 0.062 nm for Ga^3+^), Zn^2+^ ions tended to substitute the Ga^3+^ ions, making it a possible acceptor for gallium oxide. The calculated carrier concentration and the mobility of the quasi 1D-nanowires were approximately 5.3 × 10^8^ cm^−1^ and 3.5 × 10^−2^ cm^2^/V·s, respectively. Liu et al. used NH_3_ as sources to dope nitrogen into β-Ga_2_O_3_ microwires [97]. I–V curves were measured for nitrogen-doped β-Ga_2_O_3_ microwires and undoped β-Ga_2_O_3_/nitrogen-doped β-Ga_2_O_3_ microwires, respectively. P-type conductivity was deduced based on the I–V behavior of the homojunction.

In summary, melt growth of β-Ga_2_O_3_ has been established, which means that β-Ga_2_O_3_ can be cheap to prepare with a scalable size in the future. Despite the possibility of realizing that p-type conductivity still remains controversial, transparent and highly n-type conducting beta phase gallium oxides can be obtained already. Thus, β-Ga_2_O_3_ should be a promising candidate as the substrate of vertical structure LEDs.

## 3. Epitaxial Relationship between III-Nitrides and β-Ga_2_O_3_

**Figure 4:**
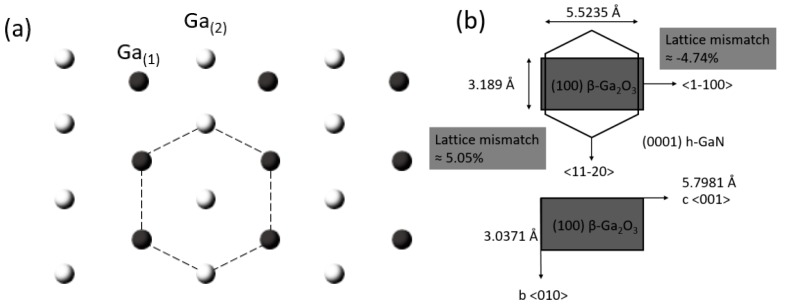
(**a**) Projection of β-Ga_2_O_3_ atomic structure perpendicular to the *a*-plane, showing the hexagonal-like arrangement of the Ga atoms bonded to O_(3)_, and (**b**) schematic illustration for the epitaxial relationship between *c*-plane h-GaN and *a*-plane β-Ga_2_O_3_.

Even though there is a significant difference in crystal structure, the epitaxy of hexagonal III-Nitrides on (1 0 0) and (−2 0 1) β-Ga_2_O_3_ is still achieved [98,99,100,101,102,103]. The epitaxial relationship between monoclinic β-Ga_2_O_3_ and the wurtzite III-Nitrides is investigated at the atomic level [45]. The (1 0 0) planes of β-Ga_2_O_3_ consist of weakly bonded oxygen atoms in O_(3)_ sites, leading to the cleavage properties of the *a*-plane. Disregarding other atoms in consideration of clarity, Figure 4a shows the projection view normal to the *a*-plane along the <2 0 1> direction where gallium atoms in Ga_(1)_ and Ga_(2)_ sites are arranged in nearly a regular hexagonal structure, making it possible to grow high crystalline quality III-Nitrides on (1 0 0) β-Ga_2_O_3_. As shown in Figure 4b, the epitaxial relationship between (0 0 0 1) GaN and (1 0 0) β-Ga_2_O_3_ substrate is <0 1 0>_Ga2O3_||<1 1 −2 0>_GaN_ and <0 0 1>_Ga2O3_ ||<−1 1 0 0>_GaN,_ determined by reflection high-energy electron diffraction (RHEED) [104]. The roughly estimated lattice mismatch between (0 0 0 1) GaN and (1 0 0) β-Ga_2_O_3_ is −4.74 % along the *c*-axis and 5.05% along the *b*-axis.

**Figure 5:**
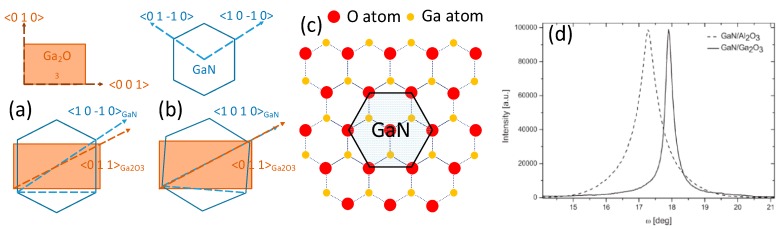
(**a**) Initial observation of epitaxial relationship between *c*-plane wurtzite GaN and (1 0 0) plane β-Ga_2_O_3_. (**b**) Refined model of epitaxial relationship between *c*-plane wurtzite GaN and (1 0 0) plane β-Ga_2_O_3_ with a stressed and reoriented GaN at the interface, the lattice mismatch (LM) minimum of 2.6% is given at a 1° tilted angle with respect to case (a). (**c**) The projection of β-Ga_2_O_3_ atomic structure perpendicular to the (−2 0 1) plane, showing the hexagonal-like arrangement of oxygen atoms. (**d**) Full width half maximum (FWHM) of HR-XRD rocking curves around the (0 0 0 2) Bragg reflection for GaN grown on Al_2_O_3_ (2580 arcsec), and on β-Ga_2_O_3_ (1020 arcsec). Reprinted with permission from Reference [105]. Copyright 2012. The Royal Society of Chemistry.

However, such an anisotropic stress state (tensile stress parallel to the *c*-axis and compressive one parallel to the *b*-axis) was considered to be of little possibility. Víllora et al. presented that there was a deviation angle of 1° calculated by image asymmetry of an electron-diffraction pattern. The model of the epitaxial relationship was adjusted, where there was a 1° titled angle compared to the former one, as illustrated in Figure 5b. The rectificatory value of lattice mismatch was then reduced to the lowest value (2.6%) ever reported [45]. As shown in Figure 5c, the oxygen atoms on (−2 0 1) planes of single crystal β-Ga_2_O_3_ are also hexagonally arranged. Thus, the deposition of hexagonal III-Nitrides is allowed. The epitaxial relationship defined as (−2 0 1)_β-Ga2O3_ || (0 0 0 1)_GaN_ with a lattice mismatch of 4.7% was presented [100]. Compared with sapphire, the lattice mismatch between III-Nitrides (GaN or AlGaN) and β-Ga_2_O_3_ is much smaller, leading to the better crystalline quality of epilayers. Kachel et al. [105] have presented that under the same condition, GaN deposited on β-Ga_2_O_3_ substrate displayed a smooth surface morphology, while that on sapphire exhibited a rough and irregular morphology. The root mean square (RMS) roughness of GaN deposited on β-Ga_2_O_3_ and sapphire was 5 nm and 75 nm, respectively. As Figure 5d shows, the full width half maximum (FWHM) of high resolution x-ray diffraction (HR-XRD) rocking curves(RCs) around the (0 0 0 2) GaN Bragg reflection peak was 1020 arcsec on β-Ga_2_O_3_, while that on Al_2_O_3_ was 2580 arcsec, manifesting a better crystalline quality using β-Ga_2_O_3_ as substrate.

## 4. Epitaxy of GaN on β-Ga_2_O_3_

### 4.1. Influences of Atmosphere

Despite the high chemical stability of β-Ga_2_O_3_, the growth of III-Nitrides (Al_x_Ga_1-x_N, x from 0 to 1) on β-Ga_2_O_3_ substrate should be carried out in a nitrogen atmosphere. As the following chemical equations show, Ga_2_O_3_ will decompose under a hydrogen atmosphere at the growth temperature of HT-GaN [106,107]:

Ga_2_O_3_ + 2H_2_ → Ga_2_O + 2H_2_O

Ga_2_O + H_2_ → 2Ga + H_2_O

**Figure 6:**
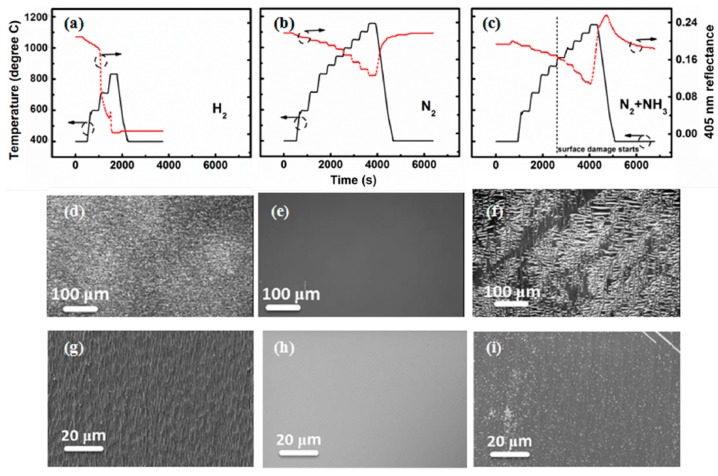
Temperature transients (temperature from EpiTT) and reflectance (405 nm) of (−2 0 1) β-Ga_2_O_3_ heated under (**a**) H_2_, (**b**) N_2_, and (**c**) N_2_ plus NH_3_ atmosphere with corresponding optical images of the resulting surfaces in (**d**), (**e**), and (**f**) and SEM images in (**g**), (**h**), and (**i**). Reprinted with permission from reference [108]. Copyright 2017 Elsevier.

Li et al. [108] heated the β-Ga_2_O_3_ substrates in a pure hydrogen, pure nitrogen, and mixed gas (98% N_2_ + 2% NH_3_) atmosphere under different temperatures including 600, 710, 830, 880, 990, 1050, 1100, and 1150 °C. Each stage was kept for 300 s. As illustrated in Figure 6a, when heated in H_2_ atmosphere, the surface reflectance of β-Ga_2_O_3_ sample dropped quickly even below 600 °C, and didn’t recover after cool down to room temperature. A decreased reflectance corresponded to a rough surface, as shown in Figure 6d,g, indicating that β-Ga_2_O_3_ was drastically damaged by H_2_. When exposed to pure nitrogen, the reflectance was first decreased with increased temperature due to the change of refractive index and recovered after cool down, manifesting that N_2_ atmosphere would not destroy the surface morphology of β-Ga_2_O_3_. The SEM images indicated a smooth surface of β-Ga_2_O_3_ after annealing, as shown in Figure 6e,h. Therefore, using N_2_ as a carrier gas was necessary for the growth of III-Nitrides in order to prevent β-Ga_2_O_3_ from decomposition. Considering that ammonia was employed as a nitrogen source for epitaxy of III-Nitrides by the metalorganic chemical vapor deposition (MOCVD) technique, 2% NH_3_ was added to the nitrogen atmosphere for heat-up process. As illustrated in Figure 6c,f,i, above 900 °C, the reflectance of β-Ga_2_O_3_ in mixed gas ambient dropped more quickly than in pure N_2_ atmosphere. It then recovered to a higher value than in pure N_2_ atmosphere when cooled down, which could be ascribed to the change of surface composition. As the following chemical equation shows, Ga_2_O_3_ would be nitrided into GaN when exposed to NH_3_ with an elevated temperature:

Ga_2_O_3_ (s) + 2NH_3_ (g) → 2GaN (s) + 3H_2_O (g)

**Figure 7:**
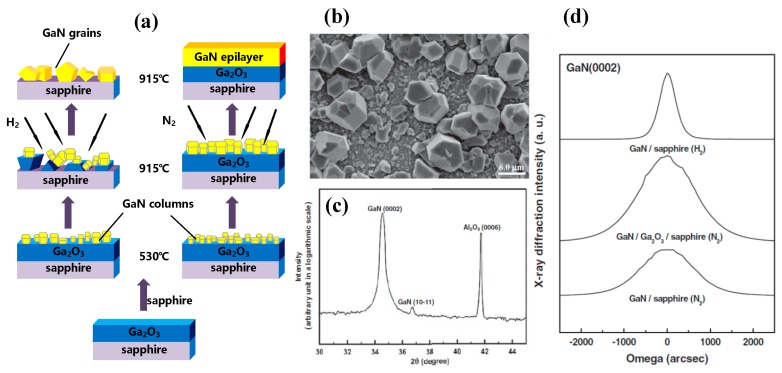
(**a**) Schematic illustration of GaN growth using N_2_ and H_2_ as carrier gases. (**b**) Top-view SEM micrograph and (**c**) double-crystal X-ray diffractometry (DC XRD) pattern of GaN deposited on Ga_2_O_3_ via MOCVD under an H_2_ atmosphere. (**d**) DC XRD of rocking curve at the (0 0 0 2) plane of the GaN/Ga_2_O_3_/sapphire fabricated under an N_2_ atmosphere and of GaN/sapphire fabricated under N_2_ and H_2_ atmospheres. Reprinted with permission from Reference [107]. Copyright 2011. The Electrochemical Society.

Figure 7a,b illustrate the grain-like, rather than film-like, surface morphology of III-Nitrides epilayers grown in H_2_ atmosphere, resulting from the etching of Ga_2_O_3_ during the epitaxy. As Figure 7c shows, the absence of Ga_2_O_3_ peaks indicate that gallium oxides will fully decompose as time goes on. However, as shown in Figure 7d, the N_2_ atmosphere will deteriorate the crystalline quality albeit instrumental for preventing Ga_2_O_3_ from decomposition [106]. It can be attributed to the different conversion and diffusion properties of the reactants under N_2_ and H_2_ atmospheres. Hillocks will generate on the GaN surface under an N_2_ atmosphere, resulting from the shorter mean free path length of reactant molecules compared to that under an H_2_ atmosphere [107]. Smaller nuclei size and coalescence thickness will be observed when exposed to N_2_ [109]. Bottcher et al. presented that threading dislocation density was inversely proportional to the average grain diameter [110]. Consequently, high density of dislocations will be generated, leading to a degraded crystalline quality of III-Nitrides epilayers. One way to obtain high crystalline quality of III-Nitrides while preventing Ga_2_O_3_ from etching is the regrowth method, which will be discussed in next section.

### 4.2. Orientations of β-Ga_2_O_3_ for Epitaxy

The epitaxy of GaN on β-Ga_2_O_3_ was initially studied on (1 0 0)-orientated substrates. Víllora et al. first demonstrated quasi-homoepitaxial growth of GaN on (1 0 0) β-Ga_2_O_3_ substrate via molecular beam epitaxy (MBE) [28]. Shimamura et al. first presented the growth of GaN on (1 0 0) β-Ga_2_O_3_ with a FWHM of 1200 arcsec by metal-organic vapor-phase epitaxy (MOVPE) [29]. Ohira et al. investigated the radio-frequency molecular beam epitaxy (RFMBE) growth of GaN on (1 0 0) β-Ga_2_O_3_ [34]. Ito et al. decreased the threading dislocation densities of GaN epilayer from 1.9 × 10^10^ cm^−2^ to 2.5 × 10^9^ cm^−2^ by utilizing a two-step growth method [45]. Kachel et al. grew GaN on (1 0 0) β-Ga_2_O_3_ by pseudo hydride vapor phase epitaxy (HVPE) method and demonstrated a self-separation method of bulk GaN from the β-Ga_2_O_3_ substrate [35]. Despite the successful deposition of GaN on β-Ga_2_O_3_ by various techniques including HVPE, MBE, and RFMBE, MOVPE is currently the most widely used method for the growth of GaN on gallium oxide. The epitaxy of GaN on β-Ga_2_O_3_ is quite different from that on sapphire due to the discrepancy of the crystal structure between wurtzite GaN and monoclinic β-Ga_2_O_3_.

**Figure 8:**
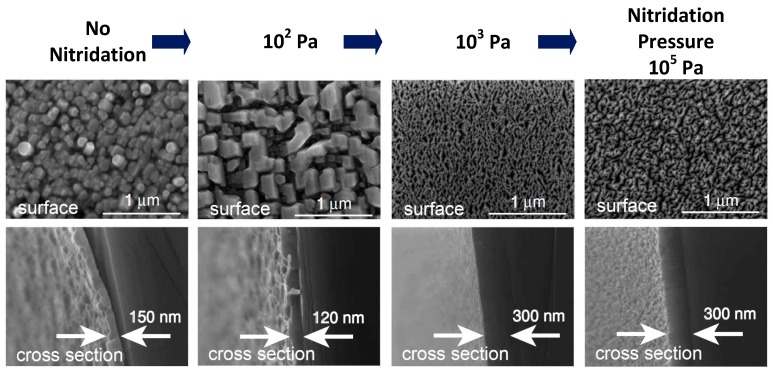
Field emission scanning electron microscope (FESEM) images of surface morphology and cross section of deposited GaN layers as a function of the nitridation pressure. Reprinted with permission from Reference [98]. Copyright 2006 Elsevier.

Prior to epitaxy, a nitridation process will reconstruct the surface of (1 0 0) β-Ga_2_O_3_ from 2-fold symmetry to 6-fold symmetry via substituting the oxygen atoms with nitrogen atoms. As a result, the quasi-homoepitaxy of (0 0 0 1) wurtzite GaN can be realized [28]. According to the first-principles density functional theory study, GaN grown on the surface modified with nitrogen is most stable [45]. Nitridation conditions have been explored by several groups [28,35]. Víllora et al. demonstrated that the nitridation pressure was one of the critical factors during this process [28]. The nitridation procedure was performed by introducing ammonia into the chamber for a certain time at the temperature of substrate close to 800 °C. Figure 8 shows the field emission scanning electron microscope (FESEM) images of surface morphology and cross section of GaN epilayers under different nitridation pressures. GaN deposited under the nitridation pressure less than 10^2^ Pa was grey and of low adhesion, indicating a non-effective nitridation process. GaN grown under pressure more than 10^3^ Pa was characterized with a mirror-like surface, suggesting a sufficient nitridation process [98]. The smooth interface indicated that the precondition would not cause a reaction in depth, but only a rearrangement on the surface [45]. A non-effective nitridation step would lead to the growth of a zincblende rather than wurtzite structure of GaN. In addition, the processing time could also determine the nitridation effect. Ohira et al. demonstrated the relationship between nitridation time and the structure of the epilayer by RHEED [104]. Before nitridation, streaky patterns indicated a smooth surface of β-Ga_2_O_3_ substrate, and patterns remained streaky after the first five minutes of processing. However, when the nitridation time further increased, GaN turned into cubic phase and into a hexagonal structure after 60 min and 90 min, respectively. That is to say, the symmetry of GaN on β-Ga_2_O_3_ substrate can be adjusted by modifying the nitridation time.

**Figure 9:**
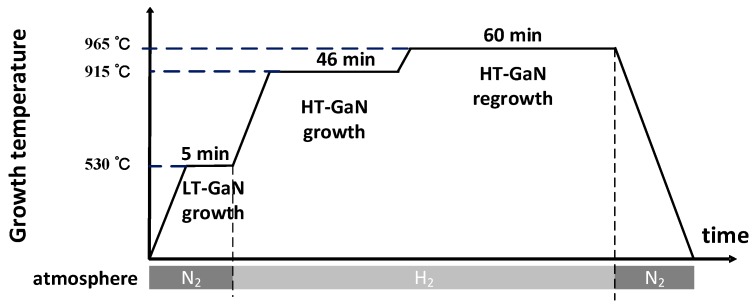
Procedure of HT-GaN regrowth method.

As mentioned before, the growth of GaN should be performed under a nitrogen atmosphere to protect Ga_2_O_3_. Tsai et al. demonstrated a regrowth method to improve the crystalline quality of epilayers that deteriorated by N_2_ atmosphere [106]. After the growth of low-temperature (LT) and high-temperature (HT) GaN under N_2_ at 530 °C and 915 °C, respectively, GaN was regrown under an H_2_ atmosphere at a temperature of 965 °C, as illustrated in Figure 9. The x-ray rocking curves (XRC) FWHM of (0 0 0 2) GaN reflection peak was decreased from 1444 arcsec to 537 arcsec. And the FWHM of photoluminescence (PL) peak was decreased from 72.5 meV to 56.7 meV. Also, the RMS roughness of the surface decreased from 30.5 nm to 0.7 nm, indicating a better crystalline quality of GaN via a regrowth method.

**Figure 10:**
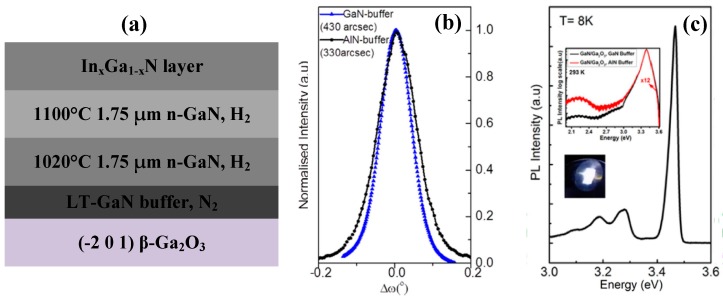
(**a**) Structural diagram of GaN on β-Ga_2_O_3_ via atmosphere switch and two-step growth. (**b**) The XRC of GaN (0 0 0 2) reflection peak for GaN grown on (−2 0 1) β-Ga_2_O_3_ substrate with GaN buffer layer and AlN buffer layer. (**c**) PL spectra of GaN grown on (−2 0 1) β-Ga_2_O_3_ substrate with a GaN buffer layer at 8 K. Reprinted with permission from Reference [103]. Copyright 2016 Springer Nature.

Previous efforts to grow GaN on *a*-plane β-Ga_2_O_3_ may be insufficient since the crystalline quality is inadequate to achieve the high-performance devices [88,89,104,105,111,112]. In addition, due to the strong cleavage properties of *a*-plane β-Ga_2_O_3_, GaN epilayer will be easily separated from β-Ga_2_O_3_ and thus complicate the process of dicing. Therefore, (−2 0 1) β-Ga_2_O_3_ as a substrate for GaN epitaxy was investigated [100,101,102,103]. The epitaxy of GaN on (−2 0 1) β-Ga_2_O_3_ with an AlN buffer layer has been reported [100,101,102], and the corresponding XRC FWHM of (0 0 0 2) GaN reflection peak was 0.122° (approximately 439 arcsec), revealing an improved crystalline quality. Muhammed et al. presented an atmosphere switch and two-step growth method. It employed N_2_ as the carrier gas for the growth of AlN buffer layer and switched it to H_2_ during the growth of HT-GaN. Flat surface morphology and high crystalline quality of GaN epilayers were obtained [102]. In addition, Muhammed et al. also demonstrated that a GaN buffer layer instead of AlN could further improve the crystalline quality of GaN epilayers [103]. By utilizing GaN buffer layer, the surface of GaN grown on Ga_2_O_3_ exhibited a homogenous strain distribution, and was nearly strain-free. The E_2_ (high) peak of GaN deposited on GaN buffer layer displayed a red shift (−0.07 cm^−1^), in contrast to that of GaN on AlN buffer layer (blue shift, 1.04 cm^−1^), suggesting a lowered strain state of GaN. Furthermore, as shown in Figure 10b,c, the FWHM of GaN (0 0 0 2) reflection peak was decreased from 430 arcsec to 330 arcsec, and the PL intensity of GaN was enhanced by a factor of 12. The calculated threading dislocation density of GaN epilayer was 1.8 (± 0.2) × 10^8^ cm^−2^ on GaN buffer layer and 4.5 (± 0.2) × 10^8^ cm^−2^ on AlN buffer layer. Consequently, the crystalline quality of a GaN epilayer was greatly improved by employing the (−2 0 1) β-Ga_2_O_3_ substrate and a GaN buffer layer. We succeeded to improve the crystal quality of GaN on gallium oxide by introducing a nanoscale epitaxial lateral overgrowth method through a self-assembled SiO_2_ nanosphere monolayer template on (−2 0 1) β-Ga_2_O_3_. Compared with direct epitaxy on β-Ga_2_O_3_, the XRC FWHM of (0 0 2) and (1 0 2) GaN reflection peak are decreased from 550.0 arcsec to 388.4 arcsec, and from 634.4 arcsec to 356.3 arcsec, respectively [113].

### 4.3. Ga_2_O_3_ Sacrificial Layer

As mentioned above, the laser lift-off technique is one way to fabricate vertical structure LEDs, but will lead to additional defects and a rough surface to devices resulting from the high energy laser irradiation [40,41,42], while chemical lift-off will not damage the interface between epilayer and substrate [43]. Recently, researches have been carried out for chemical lift-off of GaN using various interlayers [43,44,45,46,47,48,106,107,114,115,116,117,118,119,120]. Lin et al. demonstrated the CLO process of InGaN-based LEDs grown on triangle-shaped and truncated-triangle-striped patterned sapphire substrates employing AlN as a sacrificial layer [115,116]. However, using KOH as etchant might also damage the GaN grown on AlN. Horng et al. reported the vertical structure nitride LED fabricated on Cu substrate via CLO process employing an AlN/strip-patterned-SiO_2_ interlayer as a sacrificial layer [117]. In addition, the direct growth of GaN on CrN and ZnO has been demonstrated, and the CLO process to detach the GaN epilayer has been realized using CrN and ZnO as sacrificial layers [43,44,45,46,47,48]. ZnO has a lower lattice mismatch (1.6%) with GaN compared to CrN, but will decompose above 650 °C in the atmosphere of NH_3_, which is exactly the source of nitrogen in MOCVD technique.

**Figure 11:**
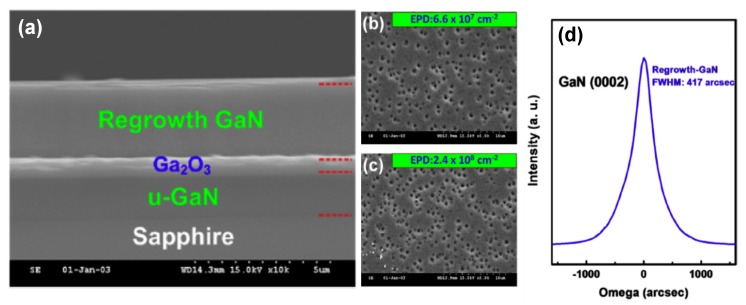
(**a**) Cross-sectional SEM image of the sample and (**b**) the etch-pit distribution observed by SEM image for the regrowth GaN epilayer grown on Ga_2_O_3_/Eco-GaN template. (**c**) The etch-pit distribution observed by SEM image for the u-GaN grown on sapphire. (**d**) XRD rocking curve of (0 0 0 2) reflection. Reprinted with permission from Reference [120]. Copyright 2015 Elsevier.

Recently, β-Ga_2_O_3_ has attracted more and more attention as the buffer layer and sacrificial layer for the CLO process of GaN due to its low lattice mismatch of 2.6% and high selectivity ratio with GaN [106,107,118,119,120]. Tsai et al. deposited a β-Ga_2_O_3_ layer on sapphire substrate via pulsed laser deposition (PLD), followed by the MOCVD growth of GaN on Ga_2_O_3_/sapphire template. The separation of GaN epilayer from sapphire by etching β-Ga_2_O_3_ away using hydrofluoric solution was demonstrated [106,118,119]. Hsueh et al. [120] reported the epitaxy of high crystalline quality GaN on MOCVD-grown Ga_2_O_3_/Eco-GaN template using O_2_ as a source of oxygen. Figure 11a shows the cross-sectional SEM image of the structure of the regrown GaN. Compared with the u-GaN grown on sapphire, the etching pits of GaN grown on a Ga_2_O_3_/Eco-GaN template were decreased from 2.4 × 10^8^ cm^−2^ to 6.6 × 10^7^ cm^−2^, as illustrated in Figure 11b,c. Employing the regrowth method mentioned above, the crystal quality of GaN was improved, with the FWHM of GaN (0 0 0 2) reflection peak reaching to 417 arcsec, as shown Figure 11d. Thus, β-Ga_2_O_3_ is a promising material acting as not only a buffer layer but also as a sacrificial for the CLO process of GaN and AlGaN epilayers with high crystalline quality. The fabrication of vertical structure LEDs can be realized by the subsequent wafer bonding process.

## 5. Epitaxy of AlGaN on β-Ga_2_O_3_

**Figure 12:**
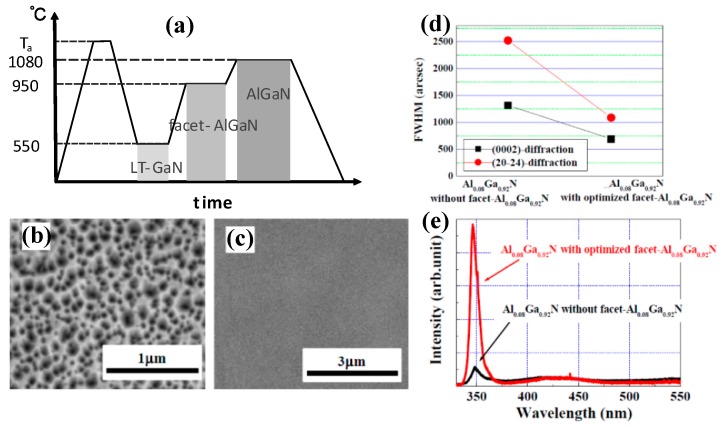
(**a**) Timing charts of growth temperatures of GaN using facet layers. Plan-view SEM images of (**b**) facet-Al_0.08_Ga_0.92_N and (**c**) Al_0.08_Ga_0.92_N grown on facet-Al_0.08_Ga_0.92_N layer at 1080 °C. (**d**) XRC FWHM and (**e**) PL spectra of Al_0.08_Ga_0.92_N without and with facet-Al_0.08_Ga_0.92_N layer. Reprinted with permission from Reference [111]. Copyright 2012 Wiley.

Although the improved crystalline quality of GaN on β-Ga_2_O_3_ substrate has been demonstrated, there is still a great challenge to grow AlGaN alloys on β-Ga_2_O_3_. Very few groups have succeeded to epitaxy AlGaN especially with high Al component alloys on β-Ga_2_O_3_. Shun Ito et al., utilizing a facet-controlled growth method, succeeded to improve the crystalline quality of Al_0.08_Ga_0.92_N epilayers [111]. Figure 12a displays the timing charts of the growth of AlGaN on a facet-AlGaN layer. Thermal annealing of (1 0 0) β-Ga_2_O_3_ substrate was performed for three minutes at 1100 °C, followed by the growth of LT-GaN buffer layer at 550 °C and 300-nm-thick facet-AlGaN layer at 950 °C. Then the deposition of AlGaN layer was carried out at 1080 °C, utilizing the MOVPE technique with trimethylaluminum, trimethylgallium, and NH_3_ as aluminum, gallium, and nitrogen sources, respectively. In addition, the deposition of LT-GaN should also be performed in nitrogen atmosphere to avoid the etching of β-Ga_2_O_3_ with H_2_. The RMS roughness of β-Ga_2_O_3_ increased from 0.2 nm to 14 nm by thermal annealing with temperature up to 1100 °C. Then the AlGaN deposited on it was characterized with inclined facets due to the rough surface of the substrate, as shown in Figure 12b. The facets distributed on the AlGaN surface were controlled by thermal annealing temperature. The dislocations produced in the facet regions bended and would not penetrate or slide to the surface, leading to lower dislocation densities (4.9 × 10^9^ cm^−2^) compared to the counterpart (2.6 × 10^10^ cm^−2^) without a facet-AlGaN layer. Moreover, as illustrated in Figure 12d, the FWHM of (0 0 0 2)- and (2 0 −2 4)-diffraction of AlGaN were decreased from approximately 2500 and 1250 arcsec to 750 arcsec and 1000 arcsec, respectively. With a facet layer, stronger PL intensity was demonstrated, indicating an improved crystalline quality of AlGaN via the facet-controlled growth method, as shown in Figure 12e.

**Figure 13:**
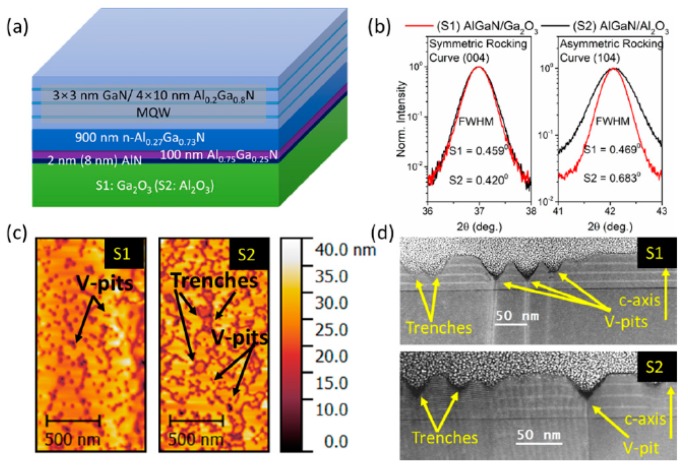
(**a**) Structural schematic of the samples. (**b**) Symmetric (0 0 4) and skew symmetric (1 0 4) XRD RCs. Reprinted with permission from reference [121]. Copyright 2018 AIP Publishing.

Recently, Ajia et al. [121] demonstrated the growth of Al_0.3_Ga_0.7_N on (−2 0 1) β-Ga_2_O_3_ by MOCVD. As shown in Figure 13a, the structure of the sample consisted of 2 nm AlN buffer deposited at 550 °C in the atmosphere of N_2_, then a 100 nm n-Al_0.75_Ga_0.25_N at 1020 °C in the atmosphere of H_2_, followed by a 900 nm n-Al_0.27_Ga_0.73_N at 1120 °C in H_2_, and ended with 3 × 3 nm GaN/4 × 10 nm Al_0.2_Ga_0.8_N multiple quantum wells (MQWs). Compared with the sample grown on sapphire, the FWHM of (1 0 4) rocking curves of the epilayer on β-Ga_2_O_3_ reduced from 0.683° to 0.469°, indicating a lower edge-type dislocation density for the sample on β-Ga_2_O_3_. A lower total density of V-pits and trenches was observed. The V-pits are typically generated from screw and mixed type of threading dislocations, while the trenches result from stacking faults [122,123]. The origin of V-pits and trenches can be ascribed to the release of strain in strained MQWs. Thus, the lower defect density of a sample grown on β-Ga_2_O_3_ manifested a relatively lower strain state compared with a sample on sapphire. Despite the demonstration of epitaxy of AlGaN on gallium oxide, the crystalline quality of epilayers are still not satisfied enough to realize high performance of UV-LEDs, which need to be improved furthermore in the future.

## 6. Vertical Structure LED

Vertical structure LEDs can be fabricated by various methods. Wang et al. employed a patterned laser lift-off process and electroplated a nickel layer for the fabrication of vertical structure GaN-based LEDs [124]. The forward voltage of VLEDs was 3.01 V and 3.39 V at 20 mA and 80 mA, respectively, which was 10% and 21% lower than conventional LEDs. With a chip size of 300 µm, a saturation current of 520 mA for VLEDs was 4.3 times higher than conventional LEDs. Also, 2.3 times and 2.7 times higher power conversion efficiency were obtained. By using the same method, Kim et al. demonstrated a 100% enhancement of light emission of GaN-VLEDs compared to lateral LEDs [125]. As reported by Lin et al., the GaN-based VLEDs prepared by laser lift-off process showed no saturation at 500 mA and a 2.7 times increase of luminance intensity [126]. Xiong et al. presented the fabrication of vertical structure GaN LEDs by wafer bonding and chemical etching lift-off GaN from Si (1 1 1) substrate. At 20 mA, the forward voltage of VLEDs was reduced to 3.2 V compared to 4.0 V of lateral LEDs. With a chip size of 240 µm, the VLEDs showed no saturation up to 800 mA, while the output power of lateral LEDs saturated at 340 mA [127]. Kawasaki et al. obtained the vertical AlGaN deep, UV-LEDs emitting 322 nm by laser lift-off technique [128]. The differential conductance of VLEDs was improved by a factor of 5. The forward voltage was half compared to the lateral LEDs. When the injection current density increased from 0 A/cm^2^ to 22 A/cm^2^, the peak wavelength of lateral UV-LED was shifted from 322 nm to 328 nm, while emission peak shift of vertical UV-LEDs was only 1 nm, indicating a reduced self-heating effect for VLEDs. Adivarahan et al. used a laser lift-off method to fabricate vertical UVLEDs with a peak emission wavelength of 280 nm. An output power of 5.5 mW of single chip devices at a continuous-wave current density of 25 A/cm^2^ was achieved [129]. Nishida et al. directly fabricated the 352 nm vertical UV-LEDs on bulk GaN [130]. An internal quantum efficiency of more than 80% and maximum output power of 10 mW were obtained. Also, vertical blue and ultraviolet LEDs on SiC have been demonstrated [131,132]. As mentioned above, lift-off technique will increase the complexity as well as the cost of the fabrication of VLEDs. Thus the direct deposition of III-Nitrides on conducting substrates is preferable. However, the high lattice and thermal mismatch between Si and III-Nitrides, the high cost of bulk GaN and SiC substrates impede their applications in VLEDs. The opaqueness of Si, GaN, and SiC in deep UV region also make them unsuitable for deep UV-LEDs. Thus, characterized with high transparency, high n-type conductivity and little lattice misfit with III-Nitrides, vertical LEDs especially UV-LEDs on β-Ga_2_O_3_ are more attractive. The relatively low cost can be expected in the future since β-Ga_2_O_3_ single crystal substrates can be prepared by melt growth.

Various researches have been carried out for VLEDs on β-Ga_2_O_3_. In 2005, Kuramata first demonstrated the InGaN/GaN based vertical LEDs (VLEDs) on β-Ga_2_O_3_ [133]. Also in 2005, Shimamura et al. achieved the blue emission from the vertical structure LEDs on conductive (1 0 0) β-Ga_2_O_3_ substrate [99]. And recently, Muhammed et al. demonstrated a high-performance vertical LED in blue region grown on (−2 0 1) β-Ga_2_O_3_ substrate via MOCVD technique [102]. The vertical GaN/InGaN LEDs on β-Ga_2_O_3_ emitting in near-UV region was first presented by Ding Li et al., with an emission wavelength around 416 nm and 410 nm on (−2 0 1) and (1 0 0) β-Ga_2_O_3_, respectively [134]. However, due to the poor crystalline quality of AlGaN, there are nearly no reports (as far as we know) in respect to the AlGaN based VLEDs on β-Ga_2_O_3_ substrate currently.

**Figure 14:**
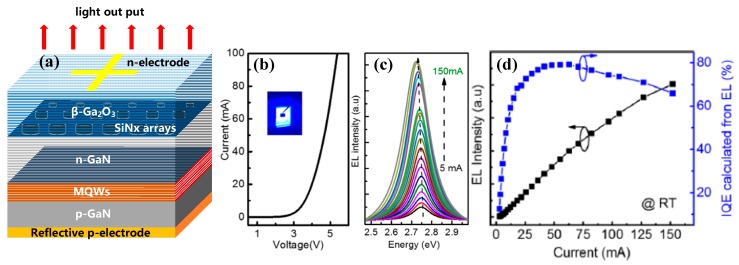
(**a**) Schematic structure of InGaN/GaN MQW vertical structure light emitting diodes (VLED). (**b**) I−V curve from InGaN/GaN MQWs VLED grown on β-Ga_2_O_3_ substrate (image of EL emission at 20 mA is shown in the inset). (**c**) EL spectra as a function of the injection current for the VLED. (**d**) EL intensity and IQE as functions of the injection current for the VLED. Reprinted with permission from reference [102]. Copyright 2017 American Chemical Society.

The common structure of InGaN/GaN based VLEDs on β-Ga_2_O_3_ is illustrated in Figure 14a. The β-Ga_2_O_3_ substrate, doped with Sn to implement the n-type conductivity (carrier density of 10^18^ cm^−3^), is masked with patterned SiN_x_ arrays. SiN_x_ arrays are used to improve the crystalline quality of epilayers via hindering the penetration of threading dislocations (TDs) to the active region, and to enhance the light extraction via decreasing the total internal reflections due to the approximation of refractive index between Ga_2_O_3_ and SiN_x_. Also, SiN_x_ arrays can reduce the resistivity between n-GaN and substrate since the interface displays a Schottky-like nature. Then a LT-AlN or LT-GaN buffer layer is deposited on β-Ga_2_O_3_. The LT-AlN (GaN) buffer layer deposited under a nitrogen atmosphere can prevent β-Ga_2_O_3_ from decomposition as well as offer the nuclei for GaN growth. Furthermore, the buffer layer also can reduce the lattice mismatch between GaN and substrate, and the thickness of buffer should be optimized. A thick buffer layer will introduce a resistivity in the interface that will degrade the vertical conductivity, while a relatively thin buffer layer lacks the ability to protect the substrates. Subsequent high-temperature GaN layer must be n-type conducting in order to achieve vertical current injection. An InGaN/GaN superlattices (SLS) layer is deposited prior to the In_x_Ga_1−x_N/GaN MQWs active region in order to uniformize the current distribution [134]. Due to the high transparency of β-Ga_2_O_3_ substrate, the backside emission from β-Ga_2_O_3_ is allowed. Finally, the highly reflective p-electrode that covers the whole area and the n-electrode with small area ratio are deposited on p-GaN layer and n-Ga_2_O_3_, respectively, which can enhance the light extraction efficiency [135]. It is worth noting that there is an inversely proportional relationship between transmittance and conductivity of β-Ga_2_O_3_. A highly transparent substrate suffers an increased resistivity, while a highly conducting one suffers a degraded transmittance. Also, intentional doping can degrade the crystalline quality and thermal conductivity of the crystals, which will deteriorate the crystalline quality of epilayers and the heat dissipation ability of devices, respectively. Thus, a moderate doping level with a carrier concentration of 10^18^ cm^−3^ may be preferable.

**Figure 15:**
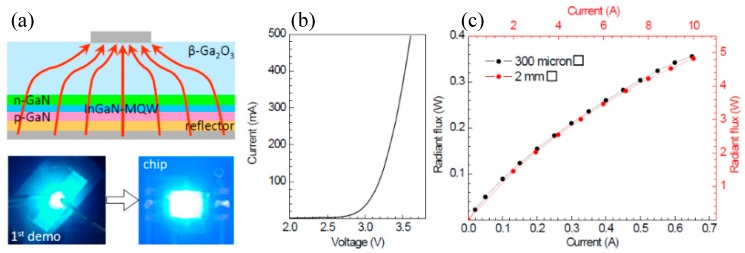
(**a**) Schematic of a blue-LED based on an InGaN-MQW deposited on a β-Ga_2_O_3_ substrate. Photograph of the initially demonstrated blue emission by vertical current injection in comparison with a current chip. (**b**) I–V characteristic of a blue-LED on β-Ga_2_O_3_ substrate. (**c**) Radiant flux as a function of the vertical current flow for two different chip areas, 300 µm (left-down black coordinates) and 2 mm (up-right red coordinates), respectively. The radiant fluxes were measured with an integrating sphere. Reprinted with permission from Reference [64]. Copyright 2014 SPIE.

VLEDs presented by Muhammed et al. displayed a turn-on voltage of 2.8 V and an operating voltage of 3.7 V under an injected current of 20 mA, as shown in Figure 14b. The low turn-on voltage and operating voltage could be ascribed to the low series resistance caused by the vertical injection of the current and the high conductivity of substrates. The reverse-bias leakage current was fully suppressed below −10 V. Moreover, bright and uniform light emission was realized, indicating a uniform current distribution. An intense blue emission at approximately 452 nm with FWHM of 97 meV was obtained under a current of 20 mA. The maximum IQE determined experimentally exceeded 78%, accompanied by a relatively small efficiency droop (∼17%) at 160 mA with respect to the maximum IQE, as illustrated in Figure 14d. The suppressed droop effect indicated a uniformized current distribution and a reduced self-heating effect of the devices. No down trend in the EL intensity existed in the range of measurement, indicating that radiative recombination was dominant and the auger recombination could be neglected at the high injection current. Due to the high quality of epilayers and the uniformity of injected current, VLEDs on β-Ga_2_O_3_ can operate at a high current without degrading the performance seriously. Thus, the saturation of VLEDs on β-Ga_2_O_3_ at a higher injection current can be expected, making it possible to achieve higher output power than conventional LEDs. The VLEDs presented by Kuramata [64] show a more uniform and bright emission compared to lateral LEDs, as illustrated in Figure 15a. It exhibited an operating voltage of 2.96 V under the current of 20 mA; with a chip size of 300 µm, a radiant flux of 360 mW at a current of 650 mA was achieved. With a chip size of 2 mm, a radiant flux of 4.82 W at a current of 10 A was obtained, as shown in Figure 15c. Even at a current density of 250 A/cm^2^, no saturation of output power was observed, indicating the possibility of realization for high-power LEDs on β-Ga_2_O_3_.

**Figure 16:**
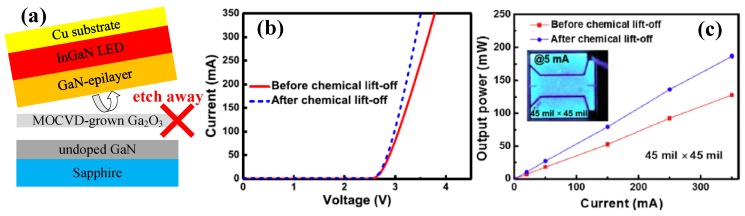
(**a**) Schematic diagram of the chemical lift-off (CLO) process for InGaN/GaN based vertical structure LEDs. (**b**) Current-voltage curves of the LED devices before and after performing the CLO process. (**c**) Light output powers as a function of the injection current for the LED devices before and after performing the CLO process. The inset shows the light emission image at 5 mA of the vertical-type LED (after CLO process) with an emission wavelength of 460 nm. Reprinted with permission from Reference [120]. Copyright 2015 Elsevier.

In addition, the fabrication of VLEDs employing β-Ga_2_O_3_ as a sacrificial layer by CLO was also demonstrated [120]. Hsueh et al. deposited a Ga_2_O_3_ layer on undoped GaN/sapphire by MOCVD, followed by the epitaxy of LED structure, and transferred it to the Cu substrate via the chemical lift-off process, as shown in Figure 16a. After chemical lift-off and the fabrication of VLED, the measured forward voltage of the device under 350 mA was reduced from 3.8 V to 3.5 V due to decreased series resistance, which could be ascribed to the reduction of the lateral current path and current crowding effect, as shown in Figure 16b. As illustrated in Figure 16c, under an injection current of 350 mA, the output power was increased from 128 mW to 187 mW after the CLO process with a chip size of 45 mil × 45 mil. The 46% enhancement of output power could be ascribed to the uniform current distribution and improved extraction efficiency for vertical structure.

As for UV-VLEDs on gallium oxide, although the PL properties have been reported, the electrical luminescence of GaN/AlGaN multiple quantum wells is still beyond realization. It can be ascribed to the poor crystalline quality of AlGaN alloys on β-Ga_2_O_3_. Due to the relatively high bond energy (2.88 eV for AlN, 2.2 eV for GaN), the high adhesion coefficient will lead to a lower mobility of Al atoms compared to Ga atoms. Unlike the layer-by-layer 2D growth mechanism of GaN, AlGaN tends to grow in a 3D islandic mode. Thus, the incorporation of Al atoms tends to occur at the initial surface positions rather than the energetically favorable sites such as steps and kinks which are easier for nucleation, leading to the formation of extended defects such as threading dislocations and grain boundaries. These defects may propagate into MQWs and can act as nonradiative centers that deteriorate the internal quantum efficiency. In addition, the high chemical activity of Al atoms will result in a pre-reaction of Al sources and N sources, which further degrade the crystalline quality of AlGaN epilayers. Therefore, it is more difficult to achieve high quality AlGaN layers on β-Ga_2_O_3_ compared to GaN. The current crystalline quality of AlGaN is not enough for the device fabrication. Despite hardly any demonstrations, it is still a promising approach to realize high-brightness and high-power for AlGaN based UV-VLEDs on β-Ga_2_O_3_ substrates due to its uniform current distribution, low series resistance, and simplified fabrication process. Compared with silicon, GaN, and SiC, the high transmittance of β-Ga_2_O_3_ in UVA and UVB spectral regions make it more attractive as a substrate of vertical UV-LEDs with an emission wavelength longer than the absorption edge (260 nm) of β-Ga_2_O_3_. In view of the mature commercialization of blue LEDs on sapphire and the dilemma of high-power UV-LEDs on sapphire, the realization of vertical UV-LEDs especially in UVB region on β-Ga_2_O_3_ is of great significance.

## 7. Conclusions

Due to its unique properties, β-Ga_2_O_3_ is a promising conductive substrate for high-performance vertical structure blue, especially UV LEDs. The epitaxial relationships between wurtzite III-Nitrides and monoclinic β-Ga_2_O_3_ were explored. Efforts to improve the crystalline quality of GaN and AlGaN epilayers have been made by trying different atmospheres, different orientations of substrates, and by utilizing various growth methods. High-quality epilayer of GaN and high-performance of blue VLEDs have been demonstrated. In the future, researches will most likely concentrate on the preparation of large-size wafers with decreased cost, the optimization of growth method, the improvement in the performance of devices, and especially the epitaxy of AlGaN materials since the advantages of β-Ga_2_O_3_ as substrates in UV-VLED are more significant. In addition, the thermal management of VLEDs on β-Ga_2_O_3_ should be carefully addressed since the thermal conductivity of gallium oxide is quite poor.
